# *catena*-Poly[[[μ-cyanido-1:2κ^2^ *C*:*N*-tricyanido-1κ^3^ *C*-bis(ethylenediamine)-2κ^4^ *N*,*N*′-copper(II)iron(II)]-μ-cyanido-κ^2^ *C*:*N*-[bis(ethylenediamine-κ^2^ *N*,*N*′)copper(II)]-μ-cyanido-κ^2^ *N*:*C*] 4.5-hy­drate]

**DOI:** 10.1107/S1600536808023830

**Published:** 2008-08-09

**Authors:** Hongling Liu, Daqi Wang

**Affiliations:** a Graduate School of New Pattern Materials Chemistry, Dezhou University, Shandong Dezhou 253023, People’s Republic of China.; b College of Chemistry and Chemical Engineering, Liaocheng University, Shandong 252059, People’s Republic of China

## Abstract

The asymmetric unit of the title compound, {[Cu_2_Fe(CN)_6_(C_2_H_8_N_2_)_4_]·4.5H_2_O}_*n*_, consists of two [Cu(C_2_H_8_N_2_)_2_]^2+^ cations, one [Fe(CN)_6_]^4−^ anion, four water mol­ecules and a half water mol­ecule that lies on a twofold rotation axis. The Fe^II^ atom is coordinated by six C atoms from three terminal and three doubly bridging CN^−^ ligands. The bridging CN^−^ ligands connect the anion to a five-coordinate [Cu(C_2_H_8_N_2_)_2_]^2+^ cation and to two symmetry-related six-coordinate [Cu(C_2_H_8_N_2_)_2_]^2+^ cations, forming a one-dimensional polymer in the *ab* plane. Inter­molecular hydrogen bonds connect the polymer units into a three-dimensional network.

## Related literature

For the corresponding complex *catena*-poly[bis­(cyanido-*C*)iron(II)]tetra(μ_2_-cyanido-*C*:*N*)bis­[bis­(ethyl­enediamine-*N*,*N′*)cadmium(II)], see: Fu & Wang (2005 ▶). For related literature, see: Fu *et al.* (2004 ▶).
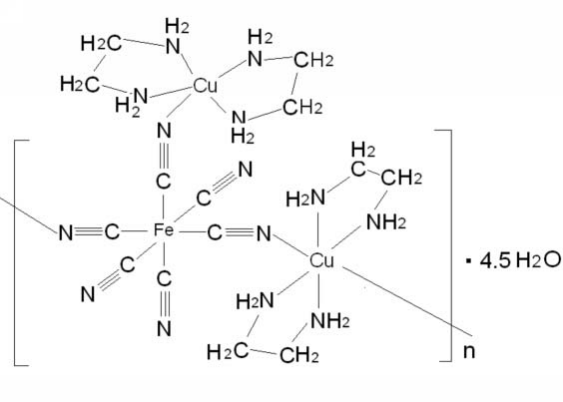


## Experimental

### 

#### Crystal data

[Cu_2_Fe(CN)_6_(C_2_H_8_N_2_)_4_]·4.5H_2_O
*M* *_r_* = 660.56
Monoclinic, 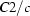

*a* = 13.481 (7) Å
*b* = 13.497 (7) Å
*c* = 31.069 (15) Å
β = 93.547 (8)°
*V* = 5642 (5) Å^3^
*Z* = 8
Mo *K*α radiation
μ = 2.05 mm^−1^
*T* = 298 (2) K
0.25 × 0.15 × 0.09 mm

#### Data collection

Bruker SMART CCD area detector diffractometer
Absorption correction: multi-scan (*SADABS*; Bruker, 1997 ▶) *T*_min_ = 0.628, *T*_max_ = 0.837
14631 measured reflections
4993 independent reflections
2395 reflections with *I* > 2σ(*I*)
*R*_int_ = 0.074

#### Refinement

*R*[*F* ^2^ > 2σ(*F* ^2^)] = 0.046
*wR*(*F* ^2^) = 0.070
*S* = 1.00
4993 reflections
348 parameters
15 restraints
H atoms treated by a mixture of independent and constrained refinement
Δρ_max_ = 0.67 e Å^−3^
Δρ_min_ = −0.47 e Å^−3^

### 

Data collection: *SMART* (Bruker, 1997 ▶); cell refinement: *SAINT* (Bruker, 1997 ▶); data reduction: *SAINT*; program(s) used to solve structure: *SHELXS97* (Sheldrick, 2008 ▶); program(s) used to refine structure: *SHELXL97* (Sheldrick, 2008 ▶); molecular graphics: *SHELXTL* (Sheldrick, 2008 ▶); software used to prepare material for publication: *SHELXTL*.

## Supplementary Material

Crystal structure: contains datablocks I, global. DOI: 10.1107/S1600536808023830/sj2512sup1.cif

Structure factors: contains datablocks I. DOI: 10.1107/S1600536808023830/sj2512Isup2.hkl

Additional supplementary materials:  crystallographic information; 3D view; checkCIF report

## Figures and Tables

**Table:** 

Cu1—N4	1.996 (4)
Cu1—N1	2.011 (5)
Cu1—N3	2.011 (4)
Cu1—N2	2.019 (4)
Cu1—N9	2.472 (5)
Cu2—N6	1.993 (4)
Cu2—N7	1.996 (4)
Cu2—N8	2.026 (4)
Cu2—N5	2.030 (4)
Cu2—N13	2.686 (5)
Fe1—C12	1.891 (6)
Fe1—C9	1.895 (6)
Fe1—C10	1.917 (5)
Fe1—C11	1.933 (6)
Fe1—C13	1.935 (6)
Fe1—C14	1.940 (6)

**Table:** 

N4—Cu1—N1	172.59 (19)
N4—Cu1—N3	84.34 (19)
N1—Cu1—N3	96.0 (2)
N4—Cu1—N2	95.6 (2)
N1—Cu1—N2	83.9 (2)
N3—Cu1—N2	178.96 (18)
N4—Cu1—N9	89.74 (17)
N1—Cu1—N9	97.67 (17)
N3—Cu1—N9	87.37 (17)
N2—Cu1—N9	93.67 (17)
N6—Cu2—N7	171.0 (2)
N6—Cu2—N8	96.40 (19)
N7—Cu2—N8	83.32 (18)
N6—Cu2—N5	83.39 (19)
N7—Cu2—N5	97.89 (18)
N8—Cu2—N5	173.55 (19)
N6—Cu2—N13	94.80 (17)
N7—Cu2—N13	94.15 (17)
N8—Cu2—N13	87.01 (16)
N5—Cu2—N13	86.58 (16)
C12—Fe1—C9	177.1 (2)
C12—Fe1—C10	92.9 (2)
C9—Fe1—C10	86.6 (2)
C12—Fe1—C11	89.5 (2)
C9—Fe1—C11	93.3 (2)
C10—Fe1—C11	88.2 (2)
C12—Fe1—C13	90.8 (2)
C9—Fe1—C13	89.8 (2)
C10—Fe1—C13	176.2 (2)
C11—Fe1—C13	90.6 (2)
C12—Fe1—C14	88.3 (2)
C9—Fe1—C14	88.9 (2)
C10—Fe1—C14	92.0 (2)
C11—Fe1—C14	177.8 (2)
C13—Fe1—C14	89.3 (2)

**Table 2:** Hydrogen-bond geometry (Å, °)

*D*—H⋯*A*	*D*—H	H⋯*A*	*D*⋯*A*	*D*—H⋯*A*
N1—H1*B*⋯O4^i^	0.90	2.59	3.316 (5)	139
N2—H2*A*⋯O2^ii^	0.90	2.14	2.999 (6)	159
N2—H2*B*⋯N14	0.90	2.57	3.335 (6)	144
N3—H3*B*⋯O5	0.90	2.18	3.074 (7)	172
N4—H4*A*⋯N10	0.90	2.63	3.308 (7)	133
N5—H5*A*⋯O3^iii^	0.90	2.33	3.214 (7)	167
N6—H6*B*⋯N9	0.90	2.17	3.044 (6)	163
N7—H7*C*⋯N12^iv^	0.90	2.55	3.293 (6)	140
N7—H7*D*⋯O5^v^	0.90	2.29	3.153 (8)	160
N8—H8*B*⋯N11^vi^	0.90	2.34	3.104 (6)	143
O1—H1⋯N11^vii^	0.81 (4)	2.01 (5)	2.807 (6)	167 (6)
O1—H2⋯N10	0.842 (14)	2.04 (3)	2.806 (6)	150 (6)
O2—H3⋯O3^viii^	0.888 (19)	1.84 (3)	2.697 (6)	161 (4)
O2—H4⋯N12^ix^	0.899 (18)	1.96 (2)	2.799 (7)	155 (2)
O3—H5⋯N14	0.873 (19)	1.88 (2)	2.741 (6)	170 (6)
O3—H6⋯N13^iii^	0.847 (19)	1.99 (2)	2.787 (6)	156 (5)
O4—H7⋯N14^iii^	0.879 (19)	2.33 (3)	3.118 (6)	149 (5)
O5—H8⋯O1^iv^	0.878 (19)	1.92 (3)	2.734 (7)	153 (5)
O5—H9⋯O2	0.839 (19)	2.09 (4)	2.844 (7)	150 (6)

Symmetry codes: (i) 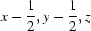; (ii) 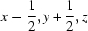; (iii) 
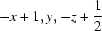
; (iv) 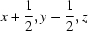; (v) 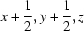; (vi) 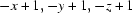; (vii) 
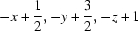
; (viii) 
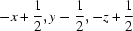
; (ix) 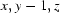.

**Fig. 1.**
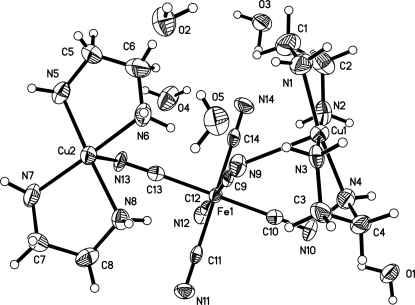
A view of the asymmetric unit of (I), with atom labels and 50% probability displacement ellipsoids.

**Fig. 2.**
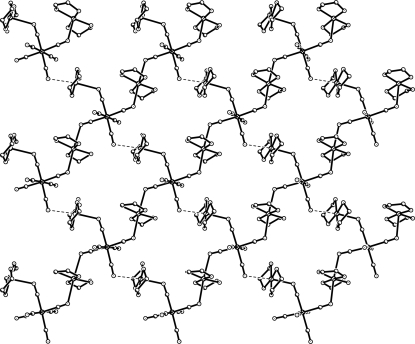
The one-dimensional polymer chains of (I) with H bonds drawn as dashed lines.

**Fig. 3.**
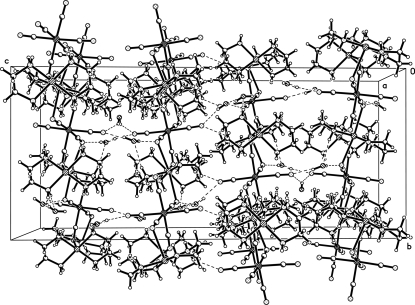
Crystal packing of (I), showing the hydrogen-bonded interactions as dashed lines.

## supplementary crystallographic information

### Comment

Hexacyanoferrate anions [Fe(CN)6]^n-^ act as good building blocks to provide bimetallic assemblies exhibiting planar structures (Fu *et al.* 2005). In this paper we report the structure of the title compouund, (I), which forms linear polymer chains.

The asymmetric unit of the title compound, [Cu_2_(C_2_H_8_N_2_)_4_Fe(CN)_6_4.5H_2_O]_n_, consists of two [Cu(C_2_H_8_N_2_]^2+^ cations, one [Fe(CN)_6_]^4-^ anion, four water molecules and a half water molecule that lies on a two-fold rotation axis (Fig. 1). The Fe1 atom is coordinated by six nitrile C atoms from three terminal CN^-^ ligands and three doubly bridging CN^-^ ligands. Cu1 is coordinated by five N atoms from two chelating ethylenediamine (en) ligands and a doubly bridging CN^1-^ ligand while Cu2 binds to six N atoms from two chelated en ligands and two doubly bridging CN^-^ ligands (Fig. 2). The average Fe—C distance of the bridging CN^1-^ ligands of 1.909 (6) Å (Table 1) is slightly shorter than that of the terminal CN^-^ ligands, 1.937 Å. The average Cu—N bond distance involving the en ligands is 2.009 Å considerably shorter than the average Cu—N(nitrile) distance of 2.472 Å. These are similar to the corresponding N—Cu bonds in the compound [Cu_2_(C_4_N_2_S_2_)_2_(C_2_H_8_N_2_)_2_]_n_ (Fu, *et al.*, 2004). The coordination geometries about the Fe1 and Cu2 centers are distorted octahedral with Cu1 dispaying a distorted square-pyramidal geometry. The bridging CN^-^ ligands connect the anion to the five coordinate Cu1 cation and to the Cu2 and Cu2^i^ (I = x+1/2, y-1/2, z) cations to form a one dimensional polymer.  In the crystal structure, the water O atoms and N atoms from the en and CN^-^ ligands participate in intermolecular hydrogen bonds (Table 2), which further connect the polymer chains into a three-dimensional network (Fig. 3).

### Experimental

A solution (10 ml) of distilled water containing CuSO_4_6H_2_O (1.0 mmol) was added slowly to aqueous mixture (20 ml)of K_4_[Fe(CN)_6_] (0.5 mmol) and NH_3_ (2 mmol). The mixture was stirred for 4 h and then filtered. crystals suitable for X-ray analysis were obtained by slow evaporation of a methanol/dichloromethane (1:2 *v*/*v*) solution over a period of three weeks. Elemental analysis found: C, 25.37%; H, 6.22%; N, 29.58%; calc. for C_28_ H_82_ Cu_4_ Fe_2_ N_28_O_9_: C, 25.45%; H, 6.26%; N, 29.69%.

### Refinement

Water H atoms were found in difference maps and were refined freely with isotropic displacement parameters. All other H atoms were placed in idealized positions and constrained to ride on their parent atoms, with C—H = 0.97 Å and *U*_iso_(H) = 1.2*U*_eq_(C), N—H = 0.90 Å, and *U*_iso_(H) = 1.2Ueq(N).

### Crystal data

**Table:**

[Cu_2_Fe(CN)_6_(C_2_H_8_N_2_)_4_]·4.5H_2_O	*F*(000) = 2744
*M**_r_* = 660.56	*D*_x_ = 1.555 Mg m^−^^3^
Monoclinic, *C*2/*c*	Mo *K*α radiation, λ = 0.71073 Å
Hall symbol: -C 2yc	Cell parameters from 1697 reflections
*a* = 13.481 (7) Å	θ = 2.3–20.2°
*b* = 13.497 (7) Å	µ = 2.05 mm^−^^1^
*c* = 31.069 (15) Å	*T* = 298 K
β = 93.547 (8)°	Block, blue
*V* = 5642 (5) Å^3^	0.25 × 0.15 × 0.09 mm
*Z* = 8	

### Data collection

**Table:**

Bruker SMART CCD area detector diffractometer	4993 independent reflections
Radiation source: fine-focus sealed tube	2395 reflections with *I* > 2σ(*I*)
graphite	*R*_int_ = 0.074
φ and ω scans	θ_max_ = 25.0°, θ_min_ = 1.3°
Absorption correction: multi-scan (*SADABS*; Bruker, 1997)	*h* = −16→16
*T*_min_ = 0.628, *T*_max_ = 0.837	*k* = −11→16
14631 measured reflections	*l* = −32→36

### Refinement

**Table:**

Refinement on *F*^2^	Primary atom site location: structure-invariant direct methods
Least-squares matrix: full	Secondary atom site location: difference Fourier map
*R*[*F*^2^ > 2σ(*F*^2^)] = 0.047	Hydrogen site location: inferred from neighbouring sites
*wR*(*F*^2^) = 0.070	H atoms treated by a mixture of independent and constrained refinement
*S* = 1.00	*w* = 1/[σ^2^(*F*_o_^2^)] where *P* = (*F*_o_^2^ + 2*F*_c_^2^)/3
4993 reflections	(Δ/σ)_max_ = 0.001
348 parameters	Δρ_max_ = 0.67 e Å^−^^3^
15 restraints	Δρ_min_ = −0.47 e Å^−^^3^

### Fractional atomic coordinates and isotropic or equivalent isotropic displacement parameters (Å^2^)

**Table:**

	*x*	*y*	*z*	*U*_iso_*/*U*_eq_	
Cu1	0.14883 (5)	0.38885 (6)	0.37321 (2)	0.0446 (2)	
Cu2	0.63210 (5)	0.39053 (6)	0.38794 (2)	0.0494 (2)	
Fe1	0.38981 (6)	0.64597 (6)	0.37999 (3)	0.0328 (2)	
N1	0.1742 (3)	0.2994 (4)	0.32332 (16)	0.0728 (17)	
H1A	0.2253	0.2584	0.3305	0.087*	
H1B	0.1200	0.2624	0.3164	0.087*	
N2	0.1342 (3)	0.4945 (3)	0.32725 (16)	0.0562 (15)	
H2A	0.0791	0.5306	0.3307	0.067*	
H2B	0.1870	0.5354	0.3292	0.067*	
N3	0.1608 (3)	0.2829 (3)	0.41874 (14)	0.0465 (13)	
H3A	0.1094	0.2405	0.4153	0.056*	
H3B	0.2175	0.2486	0.4165	0.056*	
N4	0.1050 (3)	0.4741 (3)	0.42098 (14)	0.0552 (15)	
H4A	0.1531	0.5178	0.4289	0.066*	
H4B	0.0502	0.5082	0.4120	0.066*	
N5	0.6854 (3)	0.3574 (4)	0.32995 (13)	0.0538 (14)	
H5A	0.7042	0.4135	0.3170	0.065*	
H5B	0.7390	0.3180	0.3339	0.065*	
N6	0.5073 (3)	0.3308 (3)	0.36175 (16)	0.0625 (16)	
H6A	0.4970	0.2715	0.3739	0.075*	
H6B	0.4554	0.3702	0.3668	0.075*	
N7	0.7606 (3)	0.4297 (3)	0.41861 (15)	0.0624 (16)	
H7C	0.8085	0.3861	0.4128	0.075*	
H7D	0.7791	0.4901	0.4098	0.075*	
N8	0.5739 (3)	0.4383 (3)	0.44283 (13)	0.0483 (14)	
H8A	0.5245	0.4816	0.4364	0.058*	
H8B	0.5488	0.3867	0.4569	0.058*	
N9	0.3237 (3)	0.4320 (4)	0.39355 (15)	0.0531 (15)	
N10	0.1843 (3)	0.7038 (4)	0.40890 (15)	0.0539 (15)	
N11	0.4716 (4)	0.6769 (4)	0.47419 (15)	0.0568 (16)	
N12	0.4435 (3)	0.8605 (4)	0.35978 (14)	0.0488 (14)	
N13	0.5947 (3)	0.5724 (4)	0.35587 (15)	0.0535 (16)	
N14	0.3119 (4)	0.6286 (4)	0.28505 (15)	0.0665 (18)	
O1	0.0188 (3)	0.6836 (4)	0.45869 (14)	0.0775 (16)	
O2	0.4258 (3)	0.0620 (3)	0.33942 (16)	0.0716 (14)	
O3	0.2555 (3)	0.5748 (4)	0.20204 (13)	0.0963 (19)	
O4	0.5000	0.7270 (5)	0.2500	0.099 (2)	
O5	0.3429 (3)	0.1477 (5)	0.41272 (18)	0.1026 (18)	
C1	0.1983 (5)	0.3623 (6)	0.2858 (2)	0.091 (3)	
H1C	0.2661	0.3864	0.2893	0.109*	
H1D	0.1906	0.3248	0.2591	0.109*	
C2	0.1274 (5)	0.4455 (6)	0.2852 (2)	0.086 (3)	
H2C	0.1427	0.4923	0.2629	0.104*	
H2D	0.0604	0.4211	0.2789	0.104*	
C3	0.1609 (4)	0.3306 (5)	0.46117 (18)	0.064 (2)	
H3C	0.2257	0.3589	0.4689	0.076*	
H3D	0.1456	0.2824	0.4830	0.076*	
C4	0.0834 (4)	0.4102 (5)	0.45824 (17)	0.061 (2)	
H4C	0.0177	0.3813	0.4539	0.074*	
H4D	0.0860	0.4489	0.4846	0.074*	
C5	0.6095 (5)	0.3076 (6)	0.3027 (2)	0.106 (3)	
H5C	0.6112	0.3333	0.2736	0.127*	
H5D	0.6250	0.2375	0.3018	0.127*	
C6	0.5141 (5)	0.3190 (6)	0.3164 (2)	0.110 (3)	
H6C	0.4752	0.2615	0.3071	0.132*	
H6D	0.4840	0.3763	0.3020	0.132*	
C7	0.7463 (5)	0.4309 (5)	0.46471 (19)	0.072 (2)	
H7A	0.8021	0.4630	0.4802	0.087*	
H7B	0.7407	0.3639	0.4755	0.087*	
C8	0.6534 (4)	0.4868 (5)	0.47051 (19)	0.0606 (19)	
H8C	0.6610	0.5554	0.4620	0.073*	
H8D	0.6371	0.4851	0.5005	0.073*	
C9	0.3514 (4)	0.5131 (4)	0.38950 (17)	0.0385 (16)	
C10	0.2612 (4)	0.6829 (4)	0.39777 (16)	0.0361 (15)	
C11	0.4408 (4)	0.6643 (4)	0.43898 (18)	0.0357 (16)	
C12	0.4243 (4)	0.7784 (4)	0.36780 (17)	0.0343 (15)	
C13	0.5186 (4)	0.6004 (4)	0.36404 (15)	0.0350 (14)	
C14	0.3402 (4)	0.6327 (4)	0.32031 (19)	0.0421 (16)	
H1	0.016 (4)	0.718 (5)	0.4802 (17)	0.080*	
H2	0.0778 (17)	0.678 (4)	0.4516 (17)	0.080*	
H3	0.372 (3)	0.078 (3)	0.3229 (15)	0.080*	
H4	0.4113 (17)	−0.0018 (17)	0.3443 (17)	0.080*	
H5	0.269 (4)	0.598 (4)	0.2280 (9)	0.080*	
H6	0.312 (2)	0.575 (4)	0.1913 (14)	0.080*	
H7	0.5503 (9)	0.686 (2)	0.249 (2)	0.080*	
H8	0.387 (3)	0.173 (5)	0.4315 (12)	0.080*	
H9	0.369 (4)	0.145 (5)	0.3890 (9)	0.080*	

### Atomic displacement parameters (Å^2^)

**Table:**

	*U*^11^	*U*^22^	*U*^33^	*U*^12^	*U*^13^	*U*^23^
Cu1	0.0491 (5)	0.0375 (5)	0.0478 (5)	−0.0015 (4)	0.0080 (4)	−0.0004 (4)
Cu2	0.0362 (5)	0.0676 (7)	0.0450 (5)	0.0023 (4)	0.0059 (4)	−0.0024 (4)
Fe1	0.0277 (4)	0.0340 (5)	0.0373 (5)	−0.0017 (4)	0.0067 (4)	−0.0016 (4)
N1	0.072 (4)	0.060 (4)	0.089 (5)	−0.014 (3)	0.025 (3)	−0.027 (3)
N2	0.042 (3)	0.051 (4)	0.075 (4)	−0.011 (3)	0.007 (3)	0.023 (3)
N3	0.028 (3)	0.044 (4)	0.067 (4)	−0.009 (2)	0.005 (3)	0.005 (3)
N4	0.049 (3)	0.048 (4)	0.070 (4)	−0.008 (3)	0.013 (3)	−0.014 (3)
N5	0.051 (3)	0.056 (4)	0.056 (4)	0.013 (3)	0.017 (3)	0.014 (3)
N6	0.039 (3)	0.059 (4)	0.090 (4)	0.011 (3)	0.006 (3)	−0.012 (3)
N7	0.037 (3)	0.055 (4)	0.097 (5)	0.000 (3)	0.017 (3)	−0.020 (3)
N8	0.043 (3)	0.051 (4)	0.052 (3)	0.003 (3)	0.003 (3)	0.011 (3)
N9	0.043 (3)	0.039 (4)	0.079 (4)	−0.004 (3)	0.012 (3)	0.002 (3)
N10	0.037 (3)	0.054 (4)	0.073 (4)	0.009 (3)	0.022 (3)	0.001 (3)
N11	0.062 (4)	0.076 (5)	0.032 (3)	−0.014 (3)	0.006 (3)	−0.001 (3)
N12	0.043 (3)	0.046 (4)	0.058 (4)	0.004 (3)	0.008 (2)	0.005 (3)
N13	0.029 (3)	0.067 (4)	0.066 (4)	0.007 (3)	0.020 (3)	−0.005 (3)
N14	0.063 (4)	0.099 (5)	0.036 (3)	−0.018 (3)	−0.006 (3)	−0.007 (3)
O1	0.062 (3)	0.103 (5)	0.071 (4)	−0.024 (3)	0.033 (3)	−0.032 (3)
O2	0.061 (3)	0.064 (4)	0.089 (4)	−0.001 (3)	−0.003 (3)	0.015 (3)
O3	0.064 (3)	0.175 (6)	0.052 (3)	−0.048 (4)	0.023 (3)	−0.038 (3)
O4	0.085 (5)	0.102 (7)	0.116 (6)	0.000	0.043 (6)	0.000
O5	0.068 (4)	0.124 (5)	0.115 (4)	−0.002 (4)	0.001 (3)	−0.029 (4)
C1	0.094 (6)	0.114 (8)	0.068 (6)	−0.029 (6)	0.039 (5)	−0.012 (5)
C2	0.087 (6)	0.129 (9)	0.041 (5)	−0.038 (6)	−0.010 (5)	0.023 (5)
C3	0.056 (5)	0.095 (7)	0.039 (5)	−0.018 (4)	−0.002 (4)	0.003 (4)
C4	0.060 (5)	0.080 (6)	0.046 (4)	−0.020 (4)	0.019 (4)	−0.010 (4)
C5	0.058 (5)	0.186 (10)	0.072 (6)	0.008 (6)	−0.012 (5)	−0.059 (6)
C6	0.081 (6)	0.181 (10)	0.068 (6)	−0.022 (6)	0.007 (5)	−0.053 (6)
C7	0.060 (5)	0.099 (7)	0.057 (5)	0.002 (4)	−0.012 (4)	−0.022 (4)
C8	0.072 (5)	0.059 (5)	0.054 (5)	−0.008 (4)	0.024 (4)	−0.007 (4)
C9	0.022 (3)	0.044 (5)	0.050 (4)	0.011 (3)	0.004 (3)	−0.010 (3)
C10	0.045 (4)	0.028 (4)	0.035 (4)	−0.008 (3)	0.004 (3)	0.002 (3)
C11	0.024 (3)	0.035 (4)	0.050 (4)	−0.003 (3)	0.015 (3)	0.006 (3)
C12	0.025 (3)	0.038 (4)	0.041 (4)	0.009 (3)	0.005 (3)	−0.004 (3)
C13	0.044 (4)	0.031 (4)	0.031 (3)	−0.014 (3)	0.007 (3)	0.007 (3)
C14	0.027 (4)	0.043 (4)	0.058 (4)	−0.007 (3)	0.014 (3)	0.004 (4)

### Geometric parameters (Å, °)

**Table:**

Cu1—N4	1.996 (4)	N8—C8	1.483 (6)
Cu1—N1	2.011 (5)	N8—H8A	0.9000
Cu1—N3	2.011 (4)	N8—H8B	0.9000
Cu1—N2	2.019 (4)	N9—C9	1.166 (6)
Cu1—N9	2.472 (5)	N10—C10	1.149 (5)
Cu2—N6	1.993 (4)	N11—C11	1.158 (6)
Cu2—N7	1.996 (4)	N12—C12	1.169 (6)
Cu2—N8	2.026 (4)	N13—C13	1.137 (5)
Cu2—N5	2.030 (4)	N14—C14	1.139 (6)
Cu2—N10^i^	2.686 (5)	O1—H1	0.81 (4)
Cu2—N13	2.686 (5)	O1—H2	0.842 (14)
Fe1—C12	1.891 (6)	O2—H3	0.888 (19)
Fe1—C9	1.895 (6)	O2—H4	0.899 (18)
Fe1—C10	1.917 (5)	O3—H5	0.873 (19)
Fe1—C11	1.933 (6)	O3—H6	0.847 (19)
Fe1—C13	1.935 (6)	O4—H7	0.879 (19)
Fe1—C14	1.940 (6)	O5—H8	0.878 (19)
N1—C1	1.495 (7)	O5—H9	0.839 (19)
N1—H1A	0.9000	C1—C2	1.474 (8)
N1—H1B	0.9000	C1—H1C	0.9700
N2—C2	1.463 (7)	C1—H1D	0.9700
N2—H2A	0.9000	C2—H2C	0.9700
N2—H2B	0.9000	C2—H2D	0.9700
N3—C3	1.467 (6)	C3—C4	1.497 (7)
N3—H3A	0.9000	C3—H3C	0.9700
N3—H3B	0.9000	C3—H3D	0.9700
N4—C4	1.487 (6)	C4—H4C	0.9700
N4—H4A	0.9000	C4—H4D	0.9700
N4—H4B	0.9000	C5—C6	1.388 (7)
N5—C5	1.452 (7)	C5—H5C	0.9700
N5—H5A	0.9000	C5—H5D	0.9700
N5—H5B	0.9000	C6—H6C	0.9700
N6—C6	1.427 (7)	C6—H6D	0.9700
N6—H6A	0.9000	C7—C8	1.483 (7)
N6—H6B	0.9000	C7—H7A	0.9700
N7—C7	1.457 (6)	C7—H7B	0.9700
N7—H7C	0.9000	C8—H8C	0.9700
N7—H7D	0.9000	C8—H8D	0.9700
			
N4—Cu1—N1	172.59 (19)	Cu2—N6—H6B	109.6
N4—Cu1—N3	84.34 (19)	H6A—N6—H6B	108.1
N1—Cu1—N3	96.0 (2)	C7—N7—Cu2	107.9 (3)
N4—Cu1—N2	95.6 (2)	C7—N7—H7C	110.1
N1—Cu1—N2	83.9 (2)	Cu2—N7—H7C	110.1
N3—Cu1—N2	178.96 (18)	C7—N7—H7D	110.1
N4—Cu1—N9	89.74 (17)	Cu2—N7—H7D	110.1
N1—Cu1—N9	97.67 (17)	H7C—N7—H7D	108.4
N3—Cu1—N9	87.37 (17)	C8—N8—Cu2	109.0 (3)
N2—Cu1—N9	93.67 (17)	C8—N8—H8A	109.9
N6—Cu2—N7	171.0 (2)	Cu2—N8—H8A	109.9
N6—Cu2—N8	96.40 (19)	C8—N8—H8B	109.9
N7—Cu2—N8	83.32 (18)	Cu2—N8—H8B	109.9
N6—Cu2—N5	83.39 (19)	H8A—N8—H8B	108.3
N7—Cu2—N5	97.89 (18)	C9—N9—Cu1	120.0 (4)
N8—Cu2—N5	173.55 (19)	C13—N13—Cu2	111.8 (4)
N6—Cu2—N10^i^	85.50 (17)	H1—O1—H2	110 (4)
N7—Cu2—N10^i^	85.77 (17)	H3—O2—H4	98 (2)
N8—Cu2—N10^i^	101.76 (16)	H5—O3—H6	103 (3)
N5—Cu2—N10^i^	84.66 (16)	H8—O5—H9	107 (3)
N6—Cu2—N13	94.80 (17)	C2—C1—N1	105.7 (5)
N7—Cu2—N13	94.15 (17)	C2—C1—H1C	110.6
N8—Cu2—N13	87.01 (16)	N1—C1—H1C	110.6
N5—Cu2—N13	86.58 (16)	C2—C1—H1D	110.6
N10^i^—Cu2—N13	171.15 (13)	N1—C1—H1D	110.6
C12—Fe1—C9	177.1 (2)	H1C—C1—H1D	108.7
C12—Fe1—C10	92.9 (2)	N2—C2—C1	109.2 (6)
C9—Fe1—C10	86.6 (2)	N2—C2—H2C	109.8
C12—Fe1—C11	89.5 (2)	C1—C2—H2C	109.8
C9—Fe1—C11	93.3 (2)	N2—C2—H2D	109.8
C10—Fe1—C11	88.2 (2)	C1—C2—H2D	109.8
C12—Fe1—C13	90.8 (2)	H2C—C2—H2D	108.3
C9—Fe1—C13	89.8 (2)	N3—C3—C4	107.4 (5)
C10—Fe1—C13	176.2 (2)	N3—C3—H3C	110.2
C11—Fe1—C13	90.6 (2)	C4—C3—H3C	110.2
C12—Fe1—C14	88.3 (2)	N3—C3—H3D	110.2
C9—Fe1—C14	88.9 (2)	C4—C3—H3D	110.2
C10—Fe1—C14	92.0 (2)	H3C—C3—H3D	108.5
C11—Fe1—C14	177.8 (2)	N4—C4—C3	107.1 (5)
C13—Fe1—C14	89.3 (2)	N4—C4—H4C	110.3
C1—N1—Cu1	108.4 (4)	C3—C4—H4C	110.3
C1—N1—H1A	110.0	N4—C4—H4D	110.3
Cu1—N1—H1A	110.0	C3—C4—H4D	110.3
C1—N1—H1B	110.0	H4C—C4—H4D	108.5
Cu1—N1—H1B	110.0	C6—C5—N5	113.8 (6)
H1A—N1—H1B	108.4	C6—C5—H5C	108.8
C2—N2—Cu1	108.1 (4)	N5—C5—H5C	108.8
C2—N2—H2A	110.1	C6—C5—H5D	108.8
Cu1—N2—H2A	110.1	N5—C5—H5D	108.8
C2—N2—H2B	110.1	H5C—C5—H5D	107.7
Cu1—N2—H2B	110.1	C5—C6—N6	115.6 (6)
H2A—N2—H2B	108.4	C5—C6—H6C	108.4
C3—N3—Cu1	108.4 (4)	N6—C6—H6C	108.4
C3—N3—H3A	110.0	C5—C6—H6D	108.4
Cu1—N3—H3A	110.0	N6—C6—H6D	108.4
C3—N3—H3B	110.0	H6C—C6—H6D	107.4
Cu1—N3—H3B	110.0	N7—C7—C8	106.9 (5)
H3A—N3—H3B	108.4	N7—C7—H7A	110.4
C4—N4—Cu1	109.0 (4)	C8—C7—H7A	110.4
C4—N4—H4A	109.9	N7—C7—H7B	110.4
Cu1—N4—H4A	109.9	C8—C7—H7B	110.4
C4—N4—H4B	109.9	H7A—C7—H7B	108.6
Cu1—N4—H4B	109.9	C7—C8—N8	106.9 (5)
H4A—N4—H4B	108.3	C7—C8—H8C	110.4
C5—N5—Cu2	110.1 (4)	N8—C8—H8C	110.4
C5—N5—H5A	109.6	C7—C8—H8D	110.4
Cu2—N5—H5A	109.6	N8—C8—H8D	110.4
C5—N5—H5B	109.6	H8C—C8—H8D	108.6
Cu2—N5—H5B	109.6	N9—C9—Fe1	176.3 (6)
H5A—N5—H5B	108.2	N10—C10—Fe1	178.9 (5)
C6—N6—Cu2	110.2 (4)	N11—C11—Fe1	178.9 (5)
C6—N6—H6A	109.6	N12—C12—Fe1	178.4 (5)
Cu2—N6—H6A	109.6	N13—C13—Fe1	178.0 (5)
C6—N6—H6B	109.6	N14—C14—Fe1	177.4 (6)
			
N4—Cu1—N1—C1	103.2 (16)	N1—C1—C2—N2	54.8 (7)
N3—Cu1—N1—C1	−164.2 (4)	Cu1—N3—C3—C4	42.0 (5)
N2—Cu1—N1—C1	16.8 (4)	Cu1—N4—C4—C3	39.0 (5)
N9—Cu1—N1—C1	−76.1 (4)	N3—C3—C4—N4	−53.4 (6)
N4—Cu1—N2—C2	−160.1 (4)	Cu2—N5—C5—C6	17.3 (9)
N1—Cu1—N2—C2	12.5 (4)	N5—C5—C6—N6	−29.8 (11)
N3—Cu1—N2—C2	−72 (11)	Cu2—N6—C6—C5	27.0 (9)
N9—Cu1—N2—C2	109.8 (4)	Cu2—N7—C7—C8	−47.8 (5)
N4—Cu1—N3—C3	−16.3 (4)	N7—C7—C8—N8	55.1 (6)
N1—Cu1—N3—C3	171.1 (3)	Cu2—N8—C8—C7	−35.9 (6)
N2—Cu1—N3—C3	−105 (11)	Cu1—N9—C9—Fe1	−27 (8)
N9—Cu1—N3—C3	73.7 (3)	C12—Fe1—C9—N9	−15 (11)
N1—Cu1—N4—C4	80.4 (17)	C10—Fe1—C9—N9	64 (8)
N3—Cu1—N4—C4	−12.9 (4)	C11—Fe1—C9—N9	152 (8)
N2—Cu1—N4—C4	166.0 (4)	C13—Fe1—C9—N9	−118 (8)
N9—Cu1—N4—C4	−100.3 (4)	C14—Fe1—C9—N9	−28 (8)
N6—Cu2—N5—C5	−2.0 (5)	C12—Fe1—C10—N10	−146 (33)
N7—Cu2—N5—C5	169.1 (5)	C9—Fe1—C10—N10	37 (33)
N8—Cu2—N5—C5	−90.5 (17)	C11—Fe1—C10—N10	−57 (33)
N13—Cu2—N5—C5	−97.2 (5)	C13—Fe1—C10—N10	16 (36)
N7—Cu2—N6—C6	−111.6 (13)	C14—Fe1—C10—N10	126 (33)
N8—Cu2—N6—C6	160.7 (5)	C12—Fe1—C11—N11	7(26)
N5—Cu2—N6—C6	−12.8 (5)	C9—Fe1—C11—N11	−173 (100)
N13—Cu2—N6—C6	73.2 (5)	C10—Fe1—C11—N11	−86 (26)
N6—Cu2—N7—C7	−66.9 (13)	C13—Fe1—C11—N11	97 (26)
N8—Cu2—N7—C7	21.9 (4)	C14—Fe1—C11—N11	9(30)
N5—Cu2—N7—C7	−164.5 (4)	C9—Fe1—C12—N12	34 (23)
N13—Cu2—N7—C7	108.4 (4)	C10—Fe1—C12—N12	−45 (20)
N6—Cu2—N8—C8	179.1 (4)	C11—Fe1—C12—N12	−133 (20)
N7—Cu2—N8—C8	8.1 (4)	C13—Fe1—C12—N12	136 (20)
N5—Cu2—N8—C8	−93.1 (17)	C14—Fe1—C12—N12	47 (20)
N13—Cu2—N8—C8	−86.4 (4)	Cu2—N13—C13—Fe1	41 (15)
N4—Cu1—N9—C9	−66.4 (5)	C12—Fe1—C13—N13	122 (15)
N1—Cu1—N9—C9	113.6 (5)	C9—Fe1—C13—N13	−61 (15)
N3—Cu1—N9—C9	−150.7 (5)	C10—Fe1—C13—N13	−40 (17)
N2—Cu1—N9—C9	29.3 (5)	C11—Fe1—C13—N13	32 (15)
N6—Cu2—N13—C13	53.0 (5)	C14—Fe1—C13—N13	−150 (15)
N7—Cu2—N13—C13	−126.3 (5)	C12—Fe1—C14—N14	−3(12)
N8—Cu2—N13—C13	−43.2 (5)	C9—Fe1—C14—N14	176 (100)
N5—Cu2—N13—C13	136.1 (5)	C10—Fe1—C14—N14	90 (12)
Cu1—N1—C1—C2	−42.4 (6)	C11—Fe1—C14—N14	−6(17)
Cu1—N2—C2—C1	−40.4 (6)	C13—Fe1—C14—N14	−94 (12)

Symmetry codes: (i) *x*+1/2, *y*−1/2, *z*.

### Hydrogen-bond geometry (Å, °)

**Table:**

*D*—H···*A*	*D*—H	H···*A*	*D*···*A*	*D*—H···*A*
N1—H1B···O4^ii^	0.90	2.59	3.316 (5)	139.
N2—H2A···O2^iii^	0.90	2.14	2.999 (6)	159.
N2—H2B···N14	0.90	2.57	3.335 (6)	144.
N3—H3B···O5	0.90	2.18	3.074 (7)	172.
N4—H4A···N10	0.90	2.63	3.308 (7)	133.
N5—H5A···O3^iv^	0.90	2.33	3.214 (7)	167.
N6—H6B···N9	0.90	2.17	3.044 (6)	163.
N7—H7C···N12^i^	0.90	2.55	3.293 (6)	140.
N7—H7D···O5^v^	0.90	2.29	3.153 (8)	160.
N8—H8B···N11^vi^	0.90	2.34	3.104 (6)	143.
O1—H1···N11^vii^	0.81 (4)	2.01 (5)	2.807 (6)	167 (6)
O1—H2···N10	0.84 (1)	2.04 (3)	2.806 (6)	150 (6)
O2—H3···O3^viii^	0.89 (2)	1.84 (3)	2.697 (6)	161 (4)
O2—H4···N12^ix^	0.90 (2)	1.96 (2)	2.799 (7)	155 (2)
O3—H5···N14	0.87 (2)	1.88 (2)	2.741 (6)	170 (6)
O3—H6···N13^iv^	0.85 (2)	1.99 (2)	2.787 (6)	156 (5)
O4—H7···N14^iv^	0.88 (2)	2.33 (3)	3.118 (6)	149 (5)
O5—H8···O1^i^	0.88 (2)	1.92 (3)	2.734 (7)	153 (5)
O5—H9···O2	0.84 (2)	2.09 (4)	2.844 (7)	150 (6)

Symmetry codes: (ii) *x*−1/2, *y*−1/2, *z*; (iii) *x*−1/2, *y*+1/2, *z*; (iv) −*x*+1, *y*, −*z*+1/2; (i) *x*+1/2, *y*−1/2, *z*; (v) *x*+1/2, *y*+1/2, *z*; (vi) −*x*+1, −*y*+1, −*z*+1; (vii) −*x*+1/2, −*y*+3/2, −*z*+1; (viii) −*x*+1/2, *y*−1/2, −*z*+1/2; (ix) *x*, *y*−1, *z*.
